# Estradiol Reduces Susceptibility of CD4^+^ T Cells and Macrophages to HIV-Infection

**DOI:** 10.1371/journal.pone.0062069

**Published:** 2013-04-17

**Authors:** Marta Rodriguez-Garcia, Nabanita Biswas, Mickey V. Patel, Fiona D. Barr, Sarah G. Crist, Christina Ochsenbauer, John V. Fahey, Charles R. Wira

**Affiliations:** 1 Department of Physiology and Neurobiology, Geisel School of Medicine at Dartmouth, Lebanon, New Hampshire, United States of America; 2 Department of Medicine, University of Alabama, Birmingham, Alabama, United States of America; Burnet Institute, Australia

## Abstract

The magnitude of the HIV epidemic in women requires urgent efforts to find effective preventive methods. Even though sex hormones have been described to influence HIV infection in epidemiological studies and regulate different immune responses that may affect HIV infection, the direct role that female sex hormones play in altering the susceptibility of target cells to HIV-infection is largely unknown. Here we evaluated the direct effect of 17-β-estradiol (E_2_) and ethinyl estradiol (EE) in HIV-infection of CD4^+^ T-cells and macrophages. Purified CD4^+^ T-cells and monocyte-derived macrophages were generated *in vitro* from peripheral blood and infected with R5 and X4 viruses. Treatment of CD4^+^ T-cells and macrophages with E_2_ prior to viral challenge reduced their susceptibility to HIV infection in a dose-dependent manner. Addition of E_2_ 2 h after viral challenge however did not result in reduced infection. In contrast, EE reduced infection in macrophages to a lesser extent than E_2_ and had no effect on CD4^+^ T-cell infection. Reduction of HIV-infection induced by E_2_ in CD4^+^ T-cells was not due to CCR5 down-regulation, but was an entry-mediated mechanism since infection with VSV-G pseudotyped HIV was not modified by E_2_. In macrophages, despite the lack of an effect of E_2_ on CCR5 expression, E_2_–treatment reduced viral entry 2 h after challenge and increased MIP-1β secretion. These results demonstrate the direct effect of E_2_ on susceptibility of HIV-target cells to infection and indicate that inhibition of target cell infection involves cell-entry related mechanisms.

## Introduction

Heterosexual transmission of HIV-1 remains a worldwide health challenge that is responsible for most HIV-1 transmissions to women (70–90%) [1]. Globally, young women are most vulnerable to HIV-1 infection, with rates of infection twice as high as young men, and as much as eight times higher in Sub-Saharan Africa, where women account for 59% of people living with HIV-1 [2], [3]. Furthermore, globally HIV-1 is the leading cause of death for women of reproductive age [2].

Gender discrepancies regarding HIV-1 infection and disease progression have been repeatedly reported and are due to hormonal differences among other factors [4], [5]. While plasma viral loads are lower in HIV-infected women compared to men, the rate of disease progression is greater in women [6], [7]. Additionally, sex hormone fluctuations in women have been associated with both protective and adverse effects. For example, relative to the follicular and luteal phases of the menstrual cycle, decreases in plasma viral load at ovulation, when estradiol levels are high, have been previously described [8], although others did not find any effect of the menstrual cycle on HIV-RNA levels in blood [9]. In contrast, analysis of genital secretions throughout the menstrual cycle demonstrated increased HIV-1 shedding during the luteal phase, when progesterone levels are higher, in some reports [9], [10] while others did not find any pattern of genital tract shedding during the menstrual cycle [11]. More recently, significant positive associations were found between the number of days from the luteinizing hormone surge and the number of endocervical HIV-infected cells [12]. Furthermore, serum estradiol levels in women are inversely correlated with AIDS-induced dementia [13], [14]. Additionally, studies conducted in macaques showed that intravaginal treatment with estriol for weeks prior to SIV vaginal challenge was able to protect. Protection was attributed to a cornification and thickening of the vaginal epithelia [15]. Despite the fact that associations between sex hormones and HIV-infection have been established, the underlying cellular and molecular mechanisms remain poorly understood.

17-β-estradiol (E_2_) is the main estrogen found in blood of women and exerts its actions through binding to the estrogen receptors (ER) present in the reproductive tract tissues and in immune cells in peripheral blood, including CD4^+^ T-cells and macrophages, the two main HIV-target cells [16], [17]. Binding of E_2_ to its receptors results in modulation of the expression of multiple genes. Studies by others and us illustrate the broad spectrum of actions of E_2_ on immune cells and the innate and adaptive immune response, including molecules and pathways involved in anti-viral innate immune responses [18], [19], [20]. With the exception of studies with isolated cells from the central nervous system or cell lines [21], [22], [23], very little is known about the direct effects of sex hormones on HIV-infection of immune cells. Interestingly, Asin *et al* examined the effects of sex hormones on HIV-infection and reported that different doses and combinations of estradiol and progesterone were able to regulate HIV-1 replication in peripheral blood mononuclear cells [24]. Therefore, a gap remains in our understanding of the direct effects of E_2_ in modulating susceptibility of CD4^+^ T-cells and macrophages to HIV-infection.

In this study we evaluated the effects of E_2_ on HIV infection of CD4^+^ T-cells and macrophages. We found a dose-dependent reduction of HIV-infection by E_2_ in both cell types, through a mechanism that affected the early steps of the viral cycle but was not due to CCR5 down-regulation.

## Materials and Methods

### Study Subjects

All investigations involving human subjects were conducted according to the principles expressed in the Declaration of Helsinki and carried out with the approval from the Committee for the Protection of Human Subjects (CPHS), Dartmouth Hitchcock Medical Center, and with written informed consent obtained from volunteer healthy donors recruited at Dartmouth Hitchcock Medical Center. Blood donors were anonymous, so no information regarding age or hormonal status was available and only gender information was disclosed.

### Generation of CD4^+^ T-cells and macrophages

Peripheral blood mononuclear cells (PBMC) were isolated by standard Ficoll density gradient centrifugation. To generate monocyte-derived macrophages, CD14+ cells were positively selected with magnetic beads (Miltenyi Biotech, Auburn, CA) and incubated in ultra-low attachment 6-well plates (Corning, Corning, NY) with Xvivo 15 media (Lonza, Walkersville, MD) supplemented with 10% human AB serum (Valley Biomedical, Winchester, VA) for 4 days [25]. CD4^+^ T-cells were purified from PBMC using magnetic negative selection (Miltenyi Biotech) and activated *in vitro* using the same media described above supplemented with Phytohemagglutinin (PHA) (2.5 µg/ml; Sigma, St Louis, MO) and IL-2 (50 U/ml) (AIDS Research and Reference Reagent Program, Division of AIDS, NIAID, NIH: Human rIL-2 from Dr. Maurice Gately, Hoffmann- La Roche Inc) [26] for 1 to 3 days prior to HIV-infection. Purity higher than 98% was obtained for both CD14+ cells and CD4^+^ T-cell populations after magnetic isolation (not shown).

### Hormone treatment

CD4^+^ T-cells and macrophages were treated either with 17β-estradiol (E_2_; Sigma), 17α-ethinyl estradiol (EE) or Raloxifene (Rx; Tocris Biosciences, Bristol, UK) as indicated in Results. CD4^+^ T-cells were treated during activation prior to infection (1–3 days) and/or following infection (6–7 days). Macrophages were treated during differentiation period (4 days) and/or following infection (6–7 days). For all hormone experiments E_2_, EE or Raloxifene were dissolved in 100% ethanol for an initial concentration of 1×10^−3^ M, evaporated to dryness and suspended in Xvivo 15 media (Invitrogen) containing 10% of charcoal dextran-stripped human AB serum (Valley Biomedical) to a concentration of 1×10^−5^ M. Dilutions were made to achieve final working concentrations ranging from 5×10^−8^ M to 5×10^−10^ M for E_2_ and EE. As a control, an equivalent amount of 100% ethanol without dissolved hormone was initially evaporated [27]. All control conditions contained evaporated ethanol as a control. For experiments using estrogen receptor antagonist, Raloxifene was added 30 min before E_2_ at a 100-fold excess concentration.

### Viruses

Seed stock for HIV-1_BaL_ (R5) was obtained through the AIDS Research and Reference Reagent Program, Division of AIDS, NIAID, NIH, from Dr. Suzanne Gartner, Dr. Mikulas Popovic and Dr. Robert Gallo [28]. Laboratory adapted viral strain HIV-1_IIIB_ was obtained from Dr. P. Gupta (University of Pittsburg, PA). Viral stocks were prepared by infection of PBMC activated with PHA (2.5 µg/ml and 50 U/ml IL-2) for 6-8 days as previously described [29]. Stocks were harvested when p24 concentrations reached 100 ng/ml. Titration of viral stocks was performed using PHA-stimulated PBMC as described [29]. The replication-competent GFP-encoding infectious molecular clone (pNLENG1i-BaL.ecto) [30] was derived from pNLENG1-ires [31] to express heterologous BaL *env* gene sequences in an isogenic backbone following the strategy previously described [32], [33]. This reporter virus, collectively referred to as Env-IMC-GFP, expresses GFP upon infection of HIV-1 susceptible target cells. The env-defective, GFP encoding proviral plasmid pNLENG1-ES-ires was previously described [31], was co-transfected with a VSV-G expressing plasmid to yield infectious, non-replicating pseudovirions.

### HIV-Infection

Macrophages were infected as described previously with minor modifications [25]. Briefly, macrophages were exposed to HIV-BaL for 2 h at an MOI of 0.1 and then washed to remove residual virus. CD4^+^ T-cells were infected with HIV-1 BaL or HIV-1 IIIB also for 2 h at an MOI of 0.1 and residual virus was washed away. Uninfected controls were incubated with medium without the virus for the same amount of time. After incubation, washed macrophage or CD4^+^ T-cell targets were plated in round bottom ultra low attachment 96-well plates (Corning) with or without sex hormones as indicated. Cell cultures were maintained for 6–7 days, with half of the well media collected and replaced with fresh media on day 3. Levels of p24 were measured in conditioned media by p24 ELISA (Advanced Bioscience Laboratories, Rockville, MD), and intracellularly by flow cytometry (KC57-FITC; Coulter, Danvers, MA). As a control to prove that detected p24 corresponds to de novo infection and not residual viral inocula, CD4^+^ T-cells or macrophages were incubated with Zidovudine (AZT; 10 µM) (AIDS Research and Reference Reagent Program, Division of AIDS, NIAID, NIH) during viral challenge (2 h) and throughout the post-infection period. No cytotoxicity was observed with this concentration of Zidovudine as measured by Trypan blue exclusion (Trypan Blue Solution, HyClone Laboratories, Inc; Logan, UT) at the end of the infection period.

### Flow cytometry

Prior to HIV infection, cells were stained for surface markers with the following antibody panels: The CD4+ T-cell panel included CD4, CD3 (eBioscience, San Diego, CA), CCR5 (BD Biosciences, San Jose, CA), CXCR4 (R&D, Minneapolis, MN), CD25 (Biolegend, San Diego, CA) and HLA-DR (Miltenyi Biotech). The panel for macrophages included CD14, CD4 (eBioscience), CD163 (Biolegend), CCR5 (BD Biosciences) and DC-SIGN (R&D). After infection (6–7 days), intracellular levels of p24 were analyzed. Briefly, cells were washed, fixed and permeabilized following instructions with Cytofix/Cytoperm Plus kit (BD Biosciences) for 20 min and stained for intracellular p24 with KC57-FITC antibody (Beckman Coulter) for 30 min. Analysis was performed on BD FACSCanto flow cytometer (BD Biosciences) using FACSdiva software and data were analyzed with the FlowJo software (Tree Star, Inc. Ashland, OR). Expression of surface markers was measured by the percentage of positive cells and the mean fluorescence intensity (MFI).

### RNA isolation and quantitative RT-PCR analysis

Total RNA was extracted from CD4^+^ T-cells and macrophages using the RNeasy kit (QIAGEN, Valencia, CA) with on-column DNase digestion using the RNase-Free DNase set (Qiagen). RNA was quantified and cDNA was generated with the iScript cDNA synthesis kit (Bio-Rad, Hercules, CA) as described. PCR was conducted in duplicates using the 5′ fluorogenic nuclease assay in real-time quantitative PCR using TaqMan chemistry on the ABI 7300 Prism real-time PCR instrument (Applied Biosystems, Carlsbad, CA). Primers and probe sets were obtained from Applied Biosystems assays-on-demand (GAPDH, CCR5, ER1, ER2). Amplification conditions and analysis were performed as described before [27]. Briefly, PCR was conducted using the following cycle parameters: 95°C, 12 min for 1 cycle (95°C, 20 s; 60°C, 1 min), for 40 cycles. Analysis was conducted using the sequence detection software supplied with the ABI 7300. The software calculates the threshold cycle (C_t_) for each reaction and this was used to quantify the amount of starting template in the reaction. The C_t_ values for each set of duplicate reactions were averaged for all subsequent calculations. A difference in C_t_ values (ΔC_t_) was calculated for each gene by taking the mean C_t_ of each gene of interest and subtracting the mean C_t_ for the housekeeping gene GAPDH for each cDNA sample. Relative expression levels were calculated using the formula 2^−ΔC^_t_ [34].

### ELISA

Secretion of RANTES and MIP-1β was measured in culture media from CD4^+^ T-cells and macrophages 3 days after HIV-infection by using RANTES and MIP-1β DuoSet ELISA development system (R&D, Minneapolis, MN) according to the manufacturer's instructions.

### Statistical analysis

Data were analyzed with the GraphPad Prism 5.0 software. Non-parametric test U-Mann Whitney or Wilcoxon paired test was applied for the comparison of two groups. For the comparison of three or more groups, the non-parametric Kruskal-Wallis test followed by Dunns post-test was applied. In all cases, a two sided P value <0.05 was considered statistically significant.

## Results

### 1. E_2_ reduces susceptibility of CD4^+^ T-cells to HIV-infection

To examine the direct effect of E_2_ on HIV-infection in an *in vitro* infection assay, purified CD4^+^ T-cells (>98% purity) were activated *in vitro* in the presence or absence of E_2_ for 3 days and infected with R5 (HIV-1 BaL) or X4 (IIIB) viral strains in parallel. Secreted and intracellular p24 was measured 7 days after infection as an indication of viral replication.

**Figure 1:**
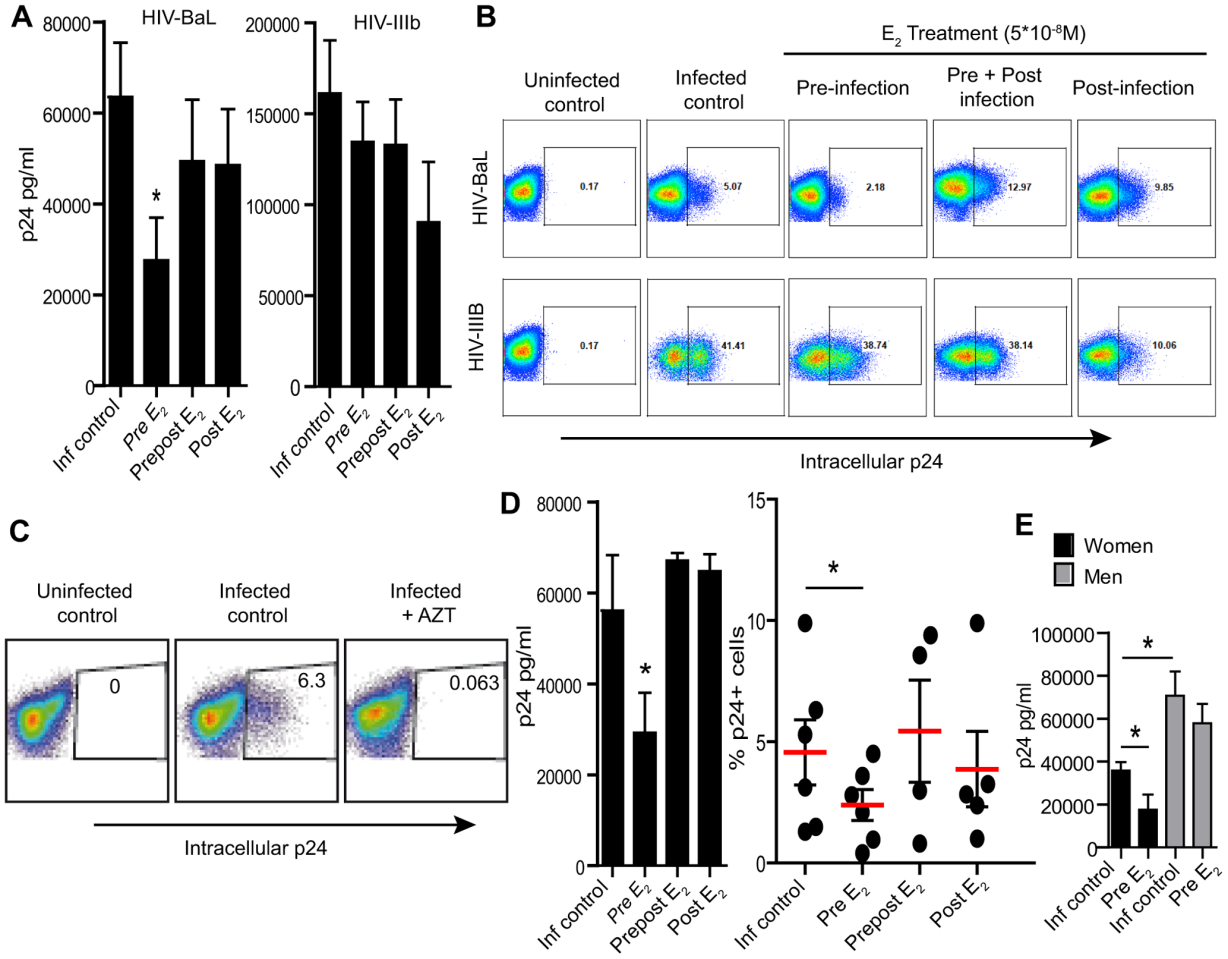
Effect of E_2_ on HIV-infection of CD4^+^ T-cells. A) Released p24 levels in the culture media after 7 days of infection when cells where pre-treated with E_2_ for 3 days (pre E_2_), treated with E_2_ before and after infection (prepost E_2_) or only after infection (post E_2_). Bars represent mean ± SEM from 7 independent experiments with different donors. *P<0.05. B) Intracellular p24 levels after 7 days of infection, representative of n = 7. C) Intracellular p24 levels after 7 days of infection in the presence of Zidovudine (AZT). D) Released p24 levels in the culture media (left panel) and intracellular p24 (right panel) after 7 days of infection when cells where pre-treated with E_2_ for 1 day (pre E_2_), treated with E_2_ before and after infection (prepost E_2_) or only after infection (post E_2_). Bars represent mean ± SEM from 6 independent experiments with different donors. *P<0.05. E) Released p24 in the culture media after 7 days of infection in CD4^+^ T-cells from women (black bars; N = 6) or men (grey bars; N = 5). *P<0.05.

As shown in Figure 1A, when CD4^+^T cells were treated with E_2_ prior to infection (pre) for 3 days, released p24 was significantly reduced 7 days after infection with HIV-1_BaL_ (56% reduction; p = 0.024). However, when E_2_ was added for the entire length of incubation (prepost) or 2 h after infection (post) no differences were found compared to the control condition. This observation was confirmed by a significant reduction in the expression of intracellular p24 in CD4^+^ T-cells pre-treated with E_2_ before infection (Figure 1B, top row). As seen in Figure 1C, addition of AZT, prevents de novo infection of CD4^+^ T-cells indicating that intracellular p24 detected is not residual inocula.

In contrast, pre-incubation of CD4^+^ T-cells with E_2_ did not reduce infection levels with HIV-1_IIIB_, measured as released p24 (Figure 1A, right panel) or intracellular p24 (Figure 1B, bottom row). As seen in Figure 1A (right side), addition of E_2_ 2 h after infection (post) slightly reduced p24 levels, but this tendency did not reach statistical significance (p = 0.077). Since no differences were found with HIV-1_IIIB_ the following experiments focused on HIV-1_BaL_.

The experiments described above were performed at a high dose of E_2_ (5×10^−8^ M), which under physiological conditions, is reached during the periovulatory phase. Since this estradiol peak lasts between 1 to 3 days, we wanted to know if exposure of CD4^+^ T-cells to a high dose of E_2_ for 1 day would be sufficient to suppress HIV-infection. Figure 1D shows that 1 day pretreatment with E_2_ significantly reduces both released and intracellular p24 levels relative to controls. Overall the same effect was observed with 3 days or 1 day of pre-treatment with E_2_ (Figures 1A and 1D respectively). Equivalent to the results obtained after activation and E_2_ treatment for 3 days (Figure 1A), only pre-incubation with E_2_ for 1 day had a suppressive effect on HIV infection, and no inhibition was measured in released or intracellular p24 (Figure 1D, left and right panels) when E_2_ was added after infection.

Since these experiments were conducted with cells from both female and male donors, we analyzed if any differences could be found between them. As shown in Figure 1E, p24 levels in the infected controls from female donors were lower than male donors, with median p24 values of 38,085 versus 71,541 respectively (p = 0.03). To determine whether E_2_ inhibited HIV infection of CD4^+^ T-cells from men and women, cells were pretreated with E_2_ for 1 day prior to infection. As seen in Figure 1E, treatment of CD4^+^ T-cells from women significantly reduced HIV-infection (54.9% reduction; p = 0.03), but had no effect on CD4^+^ T-cells from men.

We investigated whether the reduction in HlV-susceptibility induced by E_2_ could be due to differences in activation. CD4^+^ T-cells treated or not with E_2_ showed similar expression of CD25 and HLA-DR before HIV challenge (data not shown).

### 2. E_2_ reduces susceptibility of macrophages to HIV-infection

**Figure 2:**
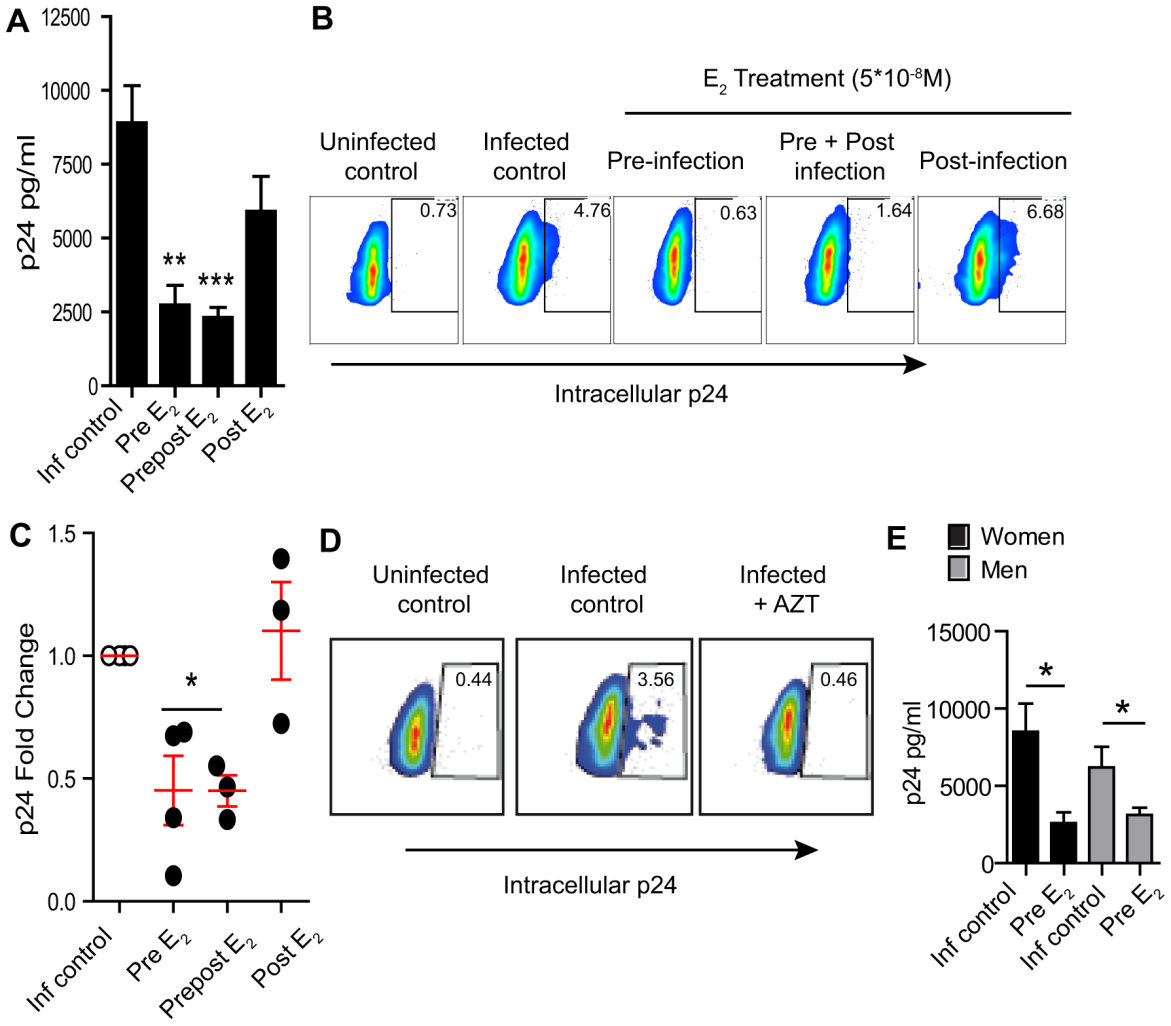
Effect of E_2_ on macrophage HIV-infection. A) Released p24 levels in the culture media after 7 days of infection when cells where pre-treated with E_2_ (pre E_2_), treated with E_2_ before and after infection (prepost E_2_) or only after infection (post E_2_). Bars represent mean ± SEM from 5 independent experiments with different donors. **P<0.01;***P<0.001. B) Representative contour plot of intracellular p24 levels after 7 days of infection and C) Fold change respect infected control of intracellular p24 from 4 different donors. Mean ± SEM is represented. *p<0.05. D) Intracellular p24 levels after 7 days of infection in the presence of Zidovudine (AZT). E) Released p24 in the culture media after 7 days of infection in CD4^+^ T-cells from women (black bars; N = 3) or men (grey bars; N = 3). *P<0.05.

Recognizing that CD4^+^ T-cells and macrophages are the most likely targets for HIV infection, we next focused on the effect of E_2_ on macrophage infection. Monocyte-derived macrophages were differentiated *in vitro* in the presence of E_2_ for 4 days and infected with HIV-1_BaL_. As shown in Figure 2A, similar to our results with CD4^+^ T-cells, viral replication was significantly reduced in macrophages differentiated in the presence of E_2_ (p<0.0003). This reduction in susceptibility to HIV-infection was induced by differentiating the macrophages in the presence of E_2_ before infection (pre; 69% reduction) and maintained when E_2_ was added back to the culture 2 h after infection (prepost; 73% reduction). In contrast, E_2_ had no effect when added immediately after infection (post) (Figure 2A). Intracellular p24 analysis confirmed a significant reduction in the percent of p24 positive cells when macrophages were differentiated in the presence of E_2_ (Figure 2B and 2C; p<0.05). Figure 2D demonstrates that intracellular p24 values correspond to de novo infection, since macrophages infected in the presence of AZT had intracellular p24 values equal to uninfected controls.

To determine whether HIV infection of macrophages was influenced by gender, blood derived macrophages from women and men were analyzed for HIV infection and responsiveness to E_2_. In contrast to CD4^+^ T-cells (Figure 1E), irrespective of donor origin, no differences in HIV infection were observed. Interestingly, infection of macrophages derived from female and male donors was significantly reduced when cells were pretreated with E_2_.

When Figures 1 and 2 are compared, E_2_ appears to be more effective in reducing susceptibility to HIV-infection in macrophages than CD4^+^ T-cells. Further, the suppressive effect was maintained when E_2_ was present after infection. This maintenance of suppression represents a difference with respect to the effect observed in CD4^+^ T-cells in that the inhibitory effect induced by pre-treatment with E_2_ is lost when E_2_ is present after infection (Figure 1).

### 3. E_2_ and ethinyl estradiol (EE) have different effects on CD4^+^ T-cell and macrophage susceptibility to HIV-infection

Recognizing that ethinyl estradiol is the main estrogenic component in most oral contraceptives and that contraceptives may be a risk factor for HIV infection [10], [35], we investigated if EE would have the same effect as E_2_ in preventing HIV-infection of target cells.

**Figure 3:**
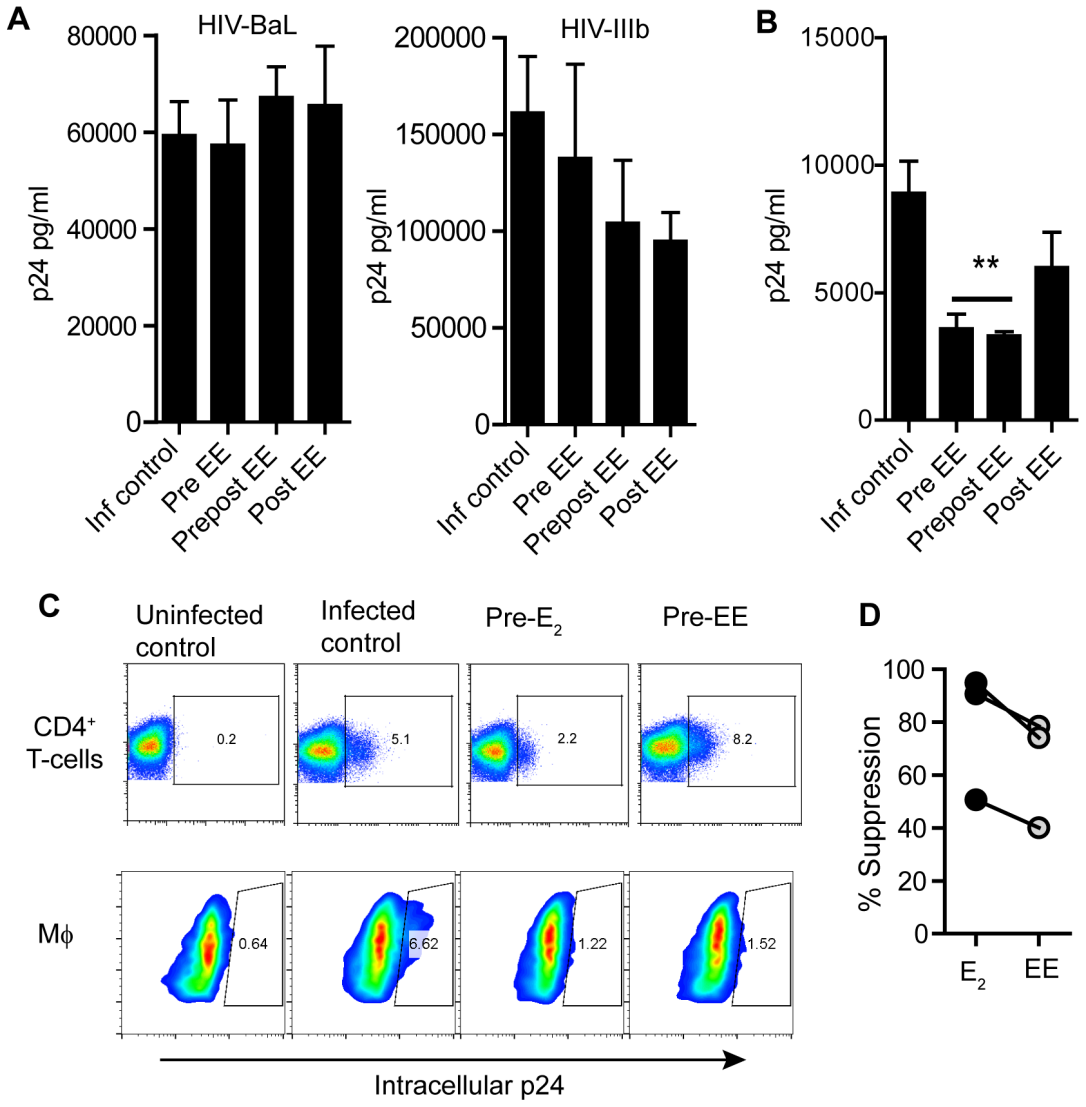
Effect of EE on HIV-infection of CD4^+^ T-cells and macrophages. A) Released p24 levels in the culture media after 7 days of infection when CD4^+^ T-cells where pre-treated with EE (pre EE), treated with EE before and after infection (prepost EE) or only after infection (post EE). Bars represent mean ± SEM from 4 independent experiments with different donors. B) Released p24 levels in the culture media after 7 days of infection when macrophages where pre-treated with EE (pre EE), treated with EE before and after infection (prepost EE) or only after infection (post EE). Bars represent mean ± SEM from 4 independent experiments with different donors. **P<0.01. C) Comparison between CD4^+^ T-cells and macrophages from the same donors pre-treated with E_2_ and EE. Dot plots represent percent of p24+ cells. D) Percent of suppression of HIV-infection respect to infected control in macrophages from the same donors pre-treated with E_2_ (black dots) or EE (grey dots) in parallel. Each dot represents one individual.

Following the same experimental design described above (Figure 1), CD4^+^ T-cells were activated in the presence of EE and infected with R5 and X4 viral strains. As seen in Figure 3A, irrespective of the time (pre) and length (prepost/post) of hormone treatment, and unlike E_2_ which inhibited HIV infection, EE had no effect on CD4^+^ T-cell infection with HIV-1_BaL_. Similar to the lack of inhibition with E_2_, we observed a pattern of reduced HIV-1_IIIb_ viral replication when EE was added 2 h after infection, but no statistical significance (Figure 3A, right panel).

In contrast, EE was able to decrease macrophage susceptibility to HIV-infection. As shown in Figure 3B, when macrophages were differentiated in the presence of EE, released p24 was significantly reduced 7 days after infection (p<0.005). When CD4^+^ T-cells and macrophages from the same donors were analyzed in parallel to compare the effects of E_2_ and EE, intracellular p24 staining confirmed the lack of effect on CD4^+^ T-cells but a decrease in the % of infected macrophages for both E_2_ and EE pre-treatments (Figure 3C). Pre-treatment with E_2_ however, was consistently 10–20% more effective than EE in suppressing viral replication (Figure 3D).

### 4. E_2_ suppresses HIV-infection in a dose-dependent manner

**Figure 4:**
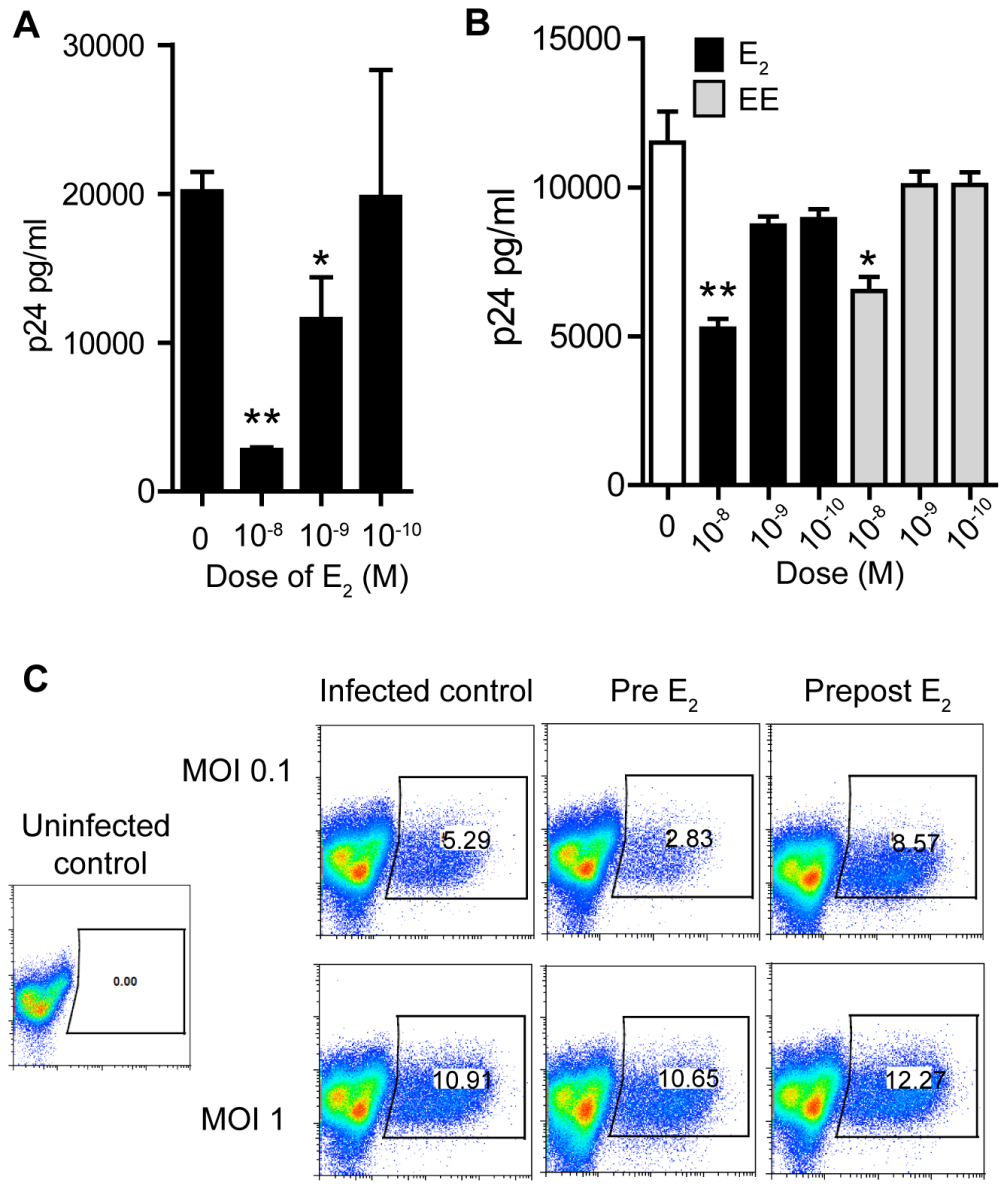
Dose response effect of E_2_ on HIV-infection. A) Released p24 levels in the culture media after 7 days of infection when CD4^+^ T-cells where pre-treated with indicated doses of E_2_. Bars represent mean ± SEM from triplicates. Representative of 3 independent experiments with different donors. *P<0.05 **P<0.01. B) Released p24 levels in the culture media after 7 days of infection when macrophages where pre-treated with indicated doses of E_2_ (black bars) or EE (grey bars). Bars represent mean ± SEM from triplicates. Representative of 3 independent experiments with different donors. *P<0.05 **P<0.01. C) Percent of GFP positive cells after infection with HIV-GFP at MOI 1 or MOI 0.1 in CD4^+^ T-cells pre-treated with E_2_ (pre E_2_) or treated before and after infection (prepost E_2_).

To determine if the observed suppression of viral replication was dose-dependent, serial 10-fold concentrations of E_2_ were tested. CD4^+^ T-cells were incubated with the different doses of E_2_ for 24 h and then infected with HIV-1_BaL_. As shown in Figure 4A, E_2_ suppressed HIV-infection of CD4^+^ T-cells in a dose-dependent fashion, with 10^−8^ M being the tested concentration that most effectively suppressed viral replication.

To evaluate the effect of E_2_ on macrophages, monocytes were differentiated into macrophages in the presence of serial 10-fold concentrations of E_2_ for 4 days and their effect on viral replication evaluated 7 days post infection. Similar to the effects observed in CD4^+^ T-cells, viral replication in macrophages was inhibited by E_2_ in a dose-dependent manner, with 10^−8^ M being the most effective concentration (Figure 4B, black bars). As a part of these studies, we also evaluated the effect of EE on macrophages and found that EE at 10^−8^ M inhibited viral replication. Consistent with our previous results, the levels of inhibition were slightly lower than those obtained with E_2_.

We then asked if the reduction in HIV-infection induced by E_2_ was dependent on the dose of viral inoculum used. To investigate this, we used replication-competent HIV reporter viruses that encodes BaL *env* and expresses GFP upon infection. CD4^+^ T-cells were treated with E_2_ for 24 h and then infected with two different concentrations of virus. As seen in Figure 4C (upper panel), infection with a MOI of 0.1 corroborated our earlier results, showing approximately a 50% reduction (5.29 to 2.83%) in number of GFP+ cells following pre-treatment with E_2_, but not when E_2_ was present both pre- and post-infection (Figure 4C, top row). In contrast, we found that at higher viral inocula (MOI 1) (Figure 4C, lower panel), the inhibitory effect of E_2_ was abrogated. These findings suggest that E_2_ induces a saturable mechanism of HIV-restriction.

### 5. E_2_ restriction of viral replication is not due to CCR5 down-regulation

**Figure 5:**
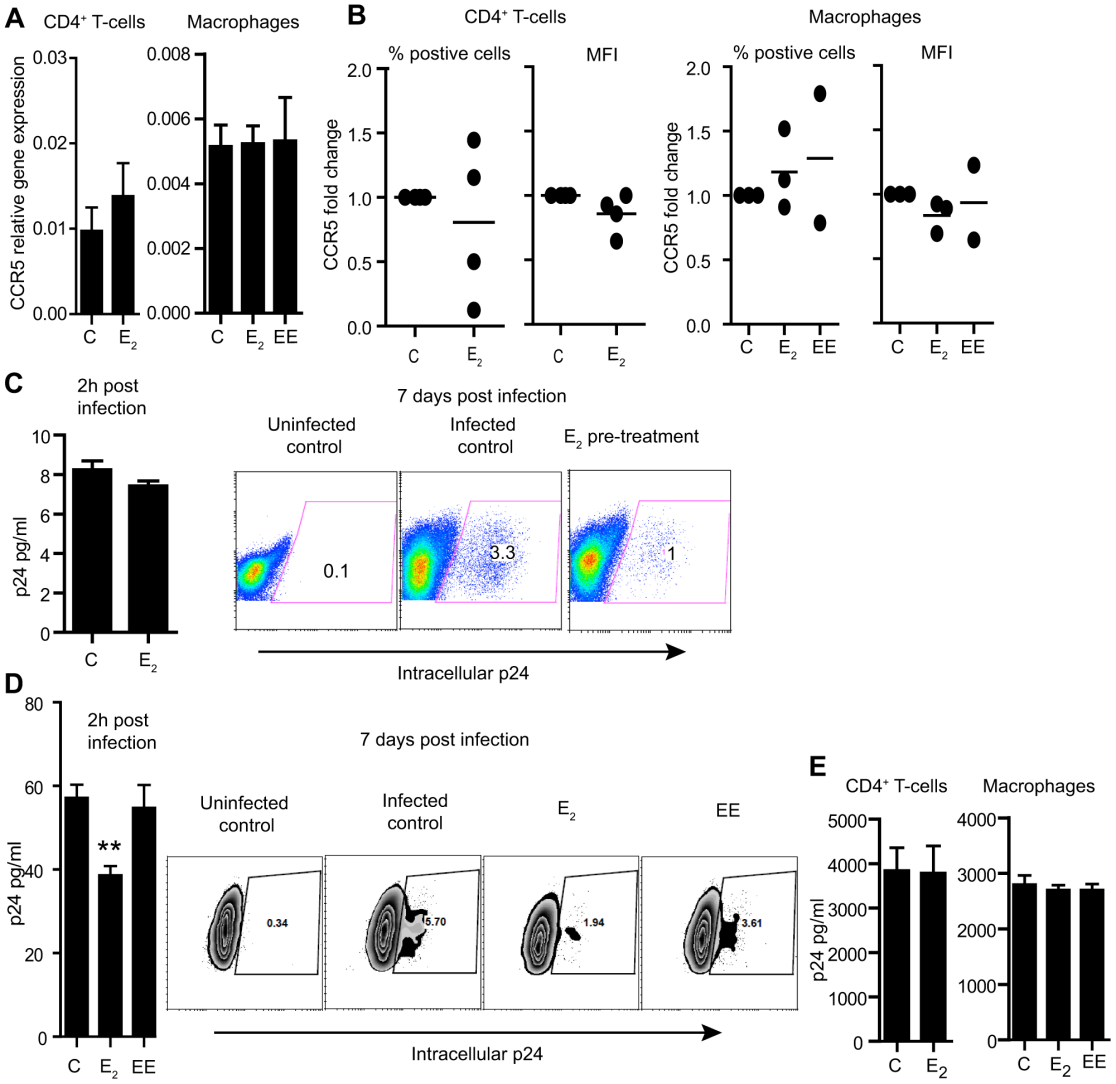
Effect of E_2_ on CCR5 expression and viral entry. A) CCR5 expression by RT-PCR. Bars represent mean ± SEM from 4 different donors. B) Surface expression of CCR5 after treatment with E_2_ or EE compared to control. C) p24 detection in cell lysates from CD4^+^ T-cells 2 h after infection (N = 4) and intracellular p24 expression 7 days after infection. D) p24 detection in cell lysates from macrophages 2 h after infection (N = 3) and intracellular p24 levels 7 days after infection. E) Released p24 in the culture media 3 days after infection with VSV-G pseudotyped HIV. Bars represent mean ± SEM from 4 independent experiments with CD4^+^ T-cells and 3 with macrophages from different donors.

To investigate the possible mechanisms involved in E_2_ suppression of viral replication, we first examined the expression of CCR5, the main coreceptor for R5 viral strains. CD4^+^ T-cells were activated in the presence of E_2_ and CCR5 expression was assayed by RNA and flow cytometry immediately prior to HIV-infection. No differences were observed in CD4^+^ T-cell CCR5 gene expression after E_2_ treatment, relative to controls (Figure 5A). When cells were analyzed for % positive cells and MFI by flow cytometry, surface expression of CCR5 in response to E_2_ was either decreased or increased depending on the donor (Figure 5B) and showed no correlation with the levels of viral suppression. Surface expression of CD4 was also measured, but no differences were found between controls and E_2_-treated cells (not shown).

To measure viral entry at 2 h post infection [36], CD4^+^ T-cells pre-treated with E_2_ for 24 h were incubated with HIV-1_BaL_ for 2 h at 37°C, after which free virus was removed by repeated washes. Cells were then trypsinized, washed and lysed prior to measuring p24 in the cell lysates by ELISA. As a control, cells were exposed to the virus for 2 h at 4°C to account for residual extracellular binding; this background was subtracted from the p24 values measured at 37°C. Half of the cells were kept in culture and HIV-infection was followed for 7 days to confirm the inhibitory activity of E_2_. As shown in Figure 5C, after 2 h of exposure to the virus, intracellular levels of p24 were not affected by E_2_ pre-treatment, but determination of infection levels 7 days after viral exposure confirmed suppression of HIV-infection by E_2_ (Figure 5C).

Macrophages differentiated in the presence of E_2_ or EE were also analyzed for the expression of CCR5 at the time of infection. As seen in Figure 5A, neither E_2_ nor EE had any effect on CCR5 gene expression relative to controls. Surface expression of CCR5 in macrophages also showed a donor variable scattered pattern after treatment with E_2_ that did not correlate with viral suppression (Figure 5B). Likewise, no differences were found regarding CD4 or DC-SIGN surface expression (not shown). Viral entry was then assessed in the same manner described above for CD4^+^ T-cells. In contrast to CD4^+^ T-cells, differentiation of macrophages in the presence of E_2_ resulted in a significant reduction of intracellular p24 after viral exposure for 2 h (Figure 5D). Interestingly, EE had no effect in reducing viral entry 2 h after viral challenge, but both E_2_ and EE reduced infection levels 7 days after (Figure 5D).

To better define the role of E_2_ in affecting viral entry, we pre-treated CD4^+^ T-cells and macrophages with E_2_ prior to infection with a single cycle, VSV-G pseudotyped virus, which enters the cells by endocytosis and bypasses receptor/coreceptor attachment and fusion [37]. As shown in Figure 5E, no differences in secreted p24 levels were found between the control cells and cells pre-treated with E_2_, strongly suggesting that E_2_ inhibits infection at the steps of viral attachment or fusion and not during reverse transcription.

### 6. Effect of E_2_ on CCR5 ligand secretion

**Figure 6:**
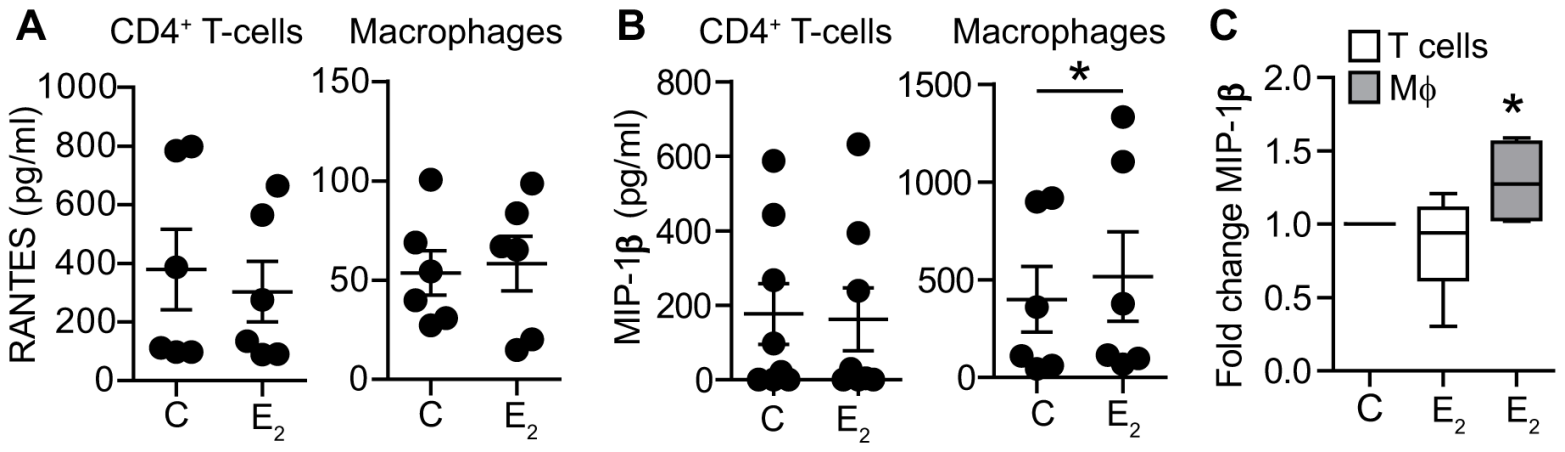
Effect of E_2_ on CCR5 ligand secretion. A) Levels of RANTES detected in the culture media from CD4^+^ T-cells (N = 6) and macrophages (N = 6) 3 days after infection with HIV-BaL. B) Levels of MIP-1β detected in culture media from CD4^+^ T-cells (N = 8) and macrophages (N = 6) 3 days after infection with HIV-BaL. Each dot represents a different donor. C) Box plot of normalized levels of MIP-1β in E_2_-treated CD4^+^ T-cells (white box) and macrophages (grey box) as compared to the control. Whiskers represent maximum and minimum values. *P<0.05.

Since our results suggest that E_2_ is affecting viral entry, without down-regulating CD4 and CCR5 expression, we explored the possibility that E_2_ treatment alters ligand secretion of RANTES and MIP-1β, known to block HIV-infection through CCR5 binding. CD4^+^ T-cells and macrophages were treated with E_2_ prior to infection with HIV_BaL_ after which secretion of RANTES and MIP-1β was analyzed. As shown in Figure 6A, E_2_ treatment had no effect on RANTES secretion by CD4^+^ T-cells or macrophages. In contrast, secretion of MIP-1β was significantly increased in macrophages (Figure 6B), but unaffected by E_2_ treatment of CD4^+^ T-cells (Figure 6C, normalized values). These findings suggest that E_2_ may reduce viral entry in macrophages by increasing MIP-1β secretion, which in turn interferes with HIV binding to CCR5 in macrophages. Whether other ligands are involved in CD4^+^ T-cell suppression on HIV infection remains to be determined.

### 7. Inhibition of HIV-infection by E_2_ is mediated through the estrogen receptor (ER)

**Figure 7:**
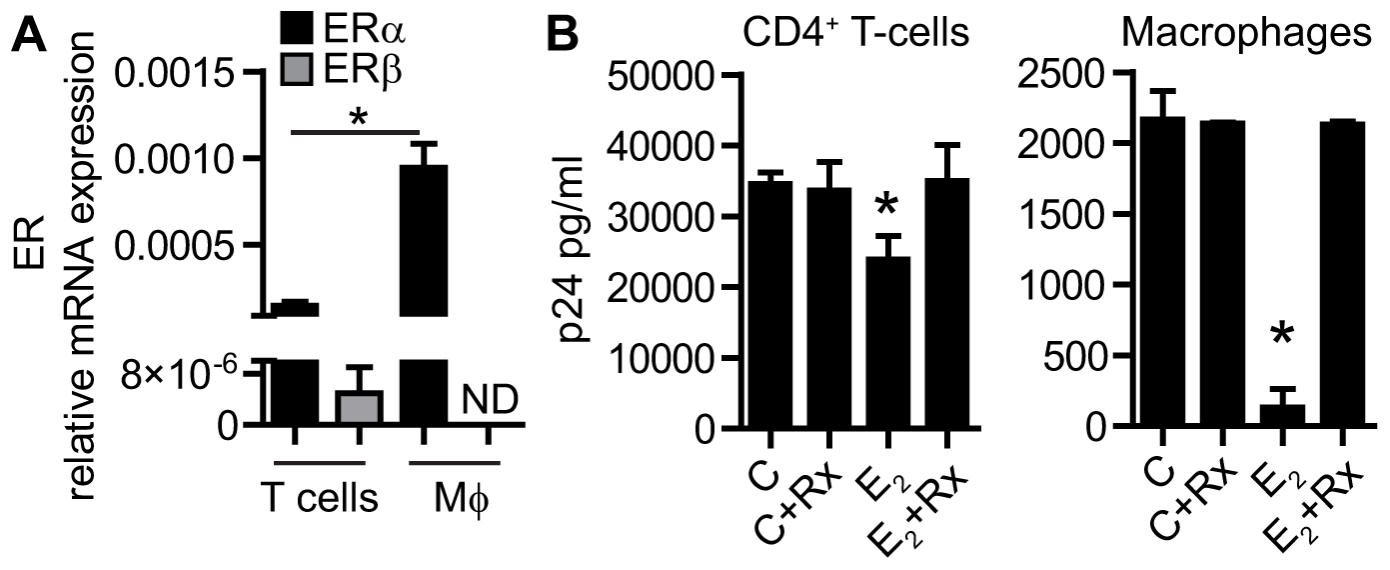
Role of estrogen receptor in E_2_-mediated suppression of HIV-infection. A) Expression of ERα (black bars) and ERβ (grey bars) detected by real time RT-PCR in CD4^+^ T-cells (N = 3) and macrophages (N = 4). Bars represent mean ± SEM from 3 and 4 different donors for CD4^+^ T-cells and macrophages respectively. ND: non-detected. B) Released p24 detected by ELISA in the culture media of CD4^+^ T-cells and macrophages after 7 days of infection. Bars represent mean ± SEM from 4 replicates. Representative of 3 independent experiments. Rx: Raloxifene. *P<0.05.

To further examine the mechanisms involved in the reduction of HIV-infection by E_2_, we investigated the role of the estrogen receptor. First we analyzed expression levels of ERα and ERβ in CD4^+^ T-cells and macrophages (Figure 7A). Both CD4^+^ T-cells and macrophages expressed ERα, with macrophages expression values approximately 10-fold higher than CD4^+^ T-cells (median expression of 0.001 vs 0.00013 respectively; p = 0.005). In contrast, ERβ expression was low compared to ERα in CD4^+^ T-cells, and undetectable in macrophages (Figure 7A). To establish that the effects of E_2_ are mediated through ER, CD4^+^ T-cells and macrophages were treated with E_2_ in the presence or not of Raloxifene, a known preferential ERα antagonist [38]. As seen in Figure 7B, when cells were pretreated with Raloxifene for 30 min prior to the addition of E_2_, Raloxifene reversed the inhibitory effects of E_2_ on p24 secretion by CD4^+^ T-cells and macrophages. Importantly, Raloxifene alone had no effect on HIV-infection.

## Discussion

In the present study we demonstrate a reduction in HIV-susceptibility of CD4^+^ T-cells and macrophages induced by E_2_. To the best of our knowledge the data presented here are the first to show that E_2_ is involved by a direct mechanism in reducing the susceptibility to HIV-infection of CD4^+^ T-cells and macrophages, the main target cells of HIV. We found that restriction of HIV-replication in target cells is not due to CCR5 down-regulation and appears to be mediated through a saturable mechanism of restriction.

Gender differences regarding HIV-infection are now well established. At the time of seroconversion women present lower viral titers than men and, compared to men at similar stages of the disease, women maintain lower viral loads [4], [5]. Despite this evidence and the known interactions between the immune system and sex hormones, little is known about the direct effect of E_2_ in modulating susceptibility of HIV-target cells to infection. A paucity of reports previously investigated the direct effects of E_2_ on HIV infection, but most focused on cells that are not the main HIV-target cells [23], [39], [40] or used cell line models that were already infected [21]. Only one previous report approached the study of PBMC [24], leaving open the question if the contribution of E_2_ was on CD4^+^ T-cells, macrophages or other cell subsets such us CD8^+^T cells or NK cells.

In our study we observed suppression of HIV-infection in CD4^+^ T-cells when they were pre-treated with E_2_ prior but not after infection. This would be in agreement with a previous study in which, when E_2_ was present throughout the experiment, no hormonal effect was observed with HIV-producing ACH-2 lymphocytes [21].

Our results also demonstrate that pre-treatment with E_2_ was able to reduce susceptibility of macrophages to HIV infection with a more pronounced effect than the one observed for CD4^+^ T-cells. Macrophages play a major role in HIV pathogenesis by contributing to HIV dissemination and establishment of viral reservoirs. As migratory cells, macrophages can reach enclosed environments such as the central nervous system that T cells cannot reach [41]. Our *in vitro* studies support the hypothesis that exposure of monocytes to high levels of E_2_ reached in peripheral blood during ovulation would render them less susceptible to HIV infection when they migrate to the tissues and differentiate into macrophages.

An unexpected finding in our study was that whereas pre-treatment with E_2_ protected CD4^+^ T-cells from HIV infection, prolonged exposure post infection, had no effect. Others have shown that the long terminal repeat of HIV contains a steroid hormone-responsive element [42] through which E_2_ enhances viral replication [22]. This could explain why addition of E_2_ before and after infection did not have the same effect as pre-treatment alone in CD4^+^ T-cells. Alternatively, others have shown cell surface expression of ERα in T cells, corresponding to the ERα46 isoform, which in response to E_2_ induces rapid phosphorylation of ERK and proliferation of T cells [43]. The plasma membrane-associated form of ER has the ability to rapidly signal in response to E_2_ and can ultimately regulate transcriptional activation [44]. These findings suggest that E_2_ added again after infection, signaling through membrane-associated ER, could mask the inhibitory effect of E_2_ pre-treatment on HIV infection. In macrophages, however, pre-treatment as well as pre- and post-treatment inhibited HIV infection while post-infection treatment with E_2_ had no effect. Just why these cells differ in their responsiveness to E_2_ remains uncertain. What is likely is that an explanation resides in the unique characteristics of macrophages given that both CD4^+^ T-cells and macrophages received the same virus. Our results indicate that CD4^+^ T-cells and macrophages display different ER expression profiles. Macrophages expressed significantly higher levels of ERα but lacked ERβ expression, while CD4^+^ T-cells expressed both forms of ER. Taking into consideration that inhibition of HIV-infection in our system is most likely mediated through ERα, as also indicated by others [45], it is possible that the differences in ER expression between cell types may account for the observed differences. Additionally, previous studies have shown that macrophages [46] and T cells [43] express different isoforms of ERα, ERα66 and ERα46, and that their relative expression is modified by E_2_ [46], [47]. Since the ERα46 isoform functions as a repressor of ERα66, differences in the expression of these isoforms will influence gene regulation [46]. The relative contribution of the different ERα isoforms to the reduction in HIV-infection induced by E_2_ remains to be elucidated. While E_2_ treatment had no effect on the internalization of virions two hours after viral challenge in CD4^+^ T-cells, macrophages differentiated in the presence of E_2_ had reduced viral entry at two hours. These findings could account for the E_2_ sustained inhibition of HIV infection in macrophages compared to CD4^+^ T-cells. As a part of these studies we investigated the early steps in HIV-infection by using a VSV-G pseudotyped virus, which does not require coreceptor-mediated attachment and fusion to enter the cells [37]. Since the viral cycle of VSV-G pseudotyped virus is the same as replicant competent HIV once the virus is inside the cell, the lack of differences in infection levels that we observed between control and E_2_-treated cells infected with VSV-G pseudotyped HIV strongly suggests that E_2_ reduces HIV-infection by affecting some step before reverse transcription. Since E_2_ had no effect on CD4 or CCR5 expression, these findings suggest that E_2_ may inhibit HIV infection by stimulating CCR5-ligand secretion, which on binding to the receptor CCR5, interferes with HIV binding to this receptor. We found a significant increase in MIP-1β secretion by E_2_-treated macrophages, but not in CD4^+^ T-cells, which could account for the differences observed between cell types. Since MIP-1β, is one of many ligands that bind CCR5, further studies are needed to more fully understand the complex mechanisms through which E_2_ functions to alter susceptibility of HIV-target cells to infection.

An interesting finding in our study was the differences in HIV-infection and responsiveness to E_2_ found in immune cells from the blood of women and men. In the absence of E_2_, CD4^+^ T-cells from women had lower levels of infection than that found in men. Additionally, CD4^+^ T-cells from women were more responsive to E_2_ than cells from men. Differential immune responsiveness to E_2_ treatment by CD4^+^ T-cells from women and men have been recently described by Moulton *et al* [48]. In their study, E_2_ increased CREMα mRNA expression and down-regulated IL-2 secretion in T cells from women more frequently than from men. In contrast to CD4^+^ T-cells, we found no significant differences between monocyte-derived macrophages from women and men. Further studies are needed to more fully define the differences in susceptibility of HIV-target cells from men and women.

Previous studies by us led to the hypothesis of a “window of vulnerability” in the menstrual cycle during which women are more susceptible to HIV infection [49]. This hypothesis was based on studies of the effects of E_2_ and progesterone on immune responses in the female reproductive tract both *in vitro* and during the menstrual cycle. The present study extends this hypothesis by demonstrating that E_2_ directly affects susceptibility of the target cells to HIV infection in a way that confers protection at a time when many aspects of the adaptive and innate immune systems are dampened, to optimize conditions for successful fertilization, implantation and pregnancy. Our dose response studies indicate that inhibition in CD4^+^ T-cells and macrophages is maximal at 5×10^−8^ M, the concentration known to saturate estrogen receptors in target cells [50]. This concentration is comparable to E_2_ levels at ovulation, and during part of the secretory phase of the menstrual cycle. Under these conditions, we hypothesize that while uterine CTL activity as well as innate protection in the cervix and vagina is suppressed [49], [51], [52], HIV-target cells would have reduced susceptibility to infection. Further studies are needed to determine whether HIV-target cell susceptibility to HIV infection varies with the menstrual cycle in the female reproductive tract (FRT). Our results are relevant to understand HIV pathogenesis, not only as a surrogate *in vitro* model for the cells present in the mucosa at the time of exposure, but also given the fact that CD4^+^ T-cell and monocyte migration from peripheral blood into the FRT is hormone and inflammation responsive. Importantly, our results suggest a protective role for E_2_ throughout the FRT at the time when fecundation is likely to occur and HIV, present in semen, is most likely to be present.

Our study indicates that ethinyl estradiol (EE), a known synthetic estrogen used in many chemical contraceptive formulations (oral, ring, etc) exerts effects on HIV-target cells that are different from those seen with E_2_, the naturally occurring estrogen. While many studies have investigated the interactions between EE and antiretroviral drugs in HIV-infected women, to the best of our knowledge, ours is the first study to evaluate the direct effect of EE in influencing susceptibility of target cells to HIV-infection. In the present study, while E_2_ had a clear effect in reducing susceptibility of CD4^+^ T-cells to HIV-infection, no significant effect was observed with EE at the same concentration. Additionally, even though EE reduced susceptibility of macrophages to HIV-infection, it was consistently less effective than E_2_. Lastly, our studies indicate that EE, unlike E_2_, played no role in reducing entry of the virus in macrophages within 2 h. These results demonstrate that EE is less effective than E_2_ in reducing susceptibility of HIV-target cells to infection. EE has a greater affinity for ER than E_2_ [50], but the mechanism(s) responsible for the differential effects between E_2_ and EE remains to be determined. As oral contraceptives are a part of multifaceted preventive strategies, these findings highlight the importance of a careful evaluation of the impact of hormonal contraception in HIV-acquisition.

In conclusion, our data indicate that E_2_ acts directly on CD4^+^ T-cells and macrophages to inhibit HIV-infection. These results should contribute to our understanding of HIV-acquisition and highlight the importance of considering the influence of menstrual cycle and hormonal contraceptives when planning new preventive strategies in women.
